# Differentially expressed genes and canonical pathways in the ascending thoracic aortic aneurysm – The Tampere Vascular Study

**DOI:** 10.1038/s41598-017-12421-4

**Published:** 2017-09-21

**Authors:** Miska Sulkava, Emma Raitoharju, Ari Mennander, Mari Levula, Ilkka Seppälä, Leo-Pekka Lyytikäinen, Otso Järvinen, Thomas Illig, Norman Klopp, Nina Mononen, Reijo Laaksonen, Mika Kähönen, Niku Oksala, Terho Lehtimäki

**Affiliations:** 1 Department of Clinical Chemistry, Fimlab Laboratories and Finnish Cardiovascular Research Center Tampere, Faculty of Medicine and Life Sciences, University of Tampere, Tampere, Finland; 2 Heart Center, Department of Cardio-Thoracic Surgery, Tampere University Hospital and University of Tampere, Faculty of Medicine and Life Sciences, Tampere, Finland; 3 Research Unit of Molecular Epidemiology, Helmholtz Zentrum, German Research Center for Environmental Health, Munich, Germany; 4 Hannover Unified Biobank, Hannover Medical School, Hannover, Germany; 5 Institute for Human Genetics, Hannover Medical School, Hanover, Germany; 6 Department of Clinical Physiology, Tampere University Hospital and University of Tampere, Faculty of Medicine and Life Sciences, Tampere, Finland

## Abstract

Ascending thoracic aortic aneurysm (ATAA) is a multifactorial disease with a strong inflammatory component. Surgery is often required to prevent aortic rupture and dissection. We performed gene expression analysis (Illumina HumanHT-12 version 3 Expression BeadChip) for 32 samples from ATAA (26 without/6 with dissection), and 28 left internal thoracic arteries (controls) collected in Tampere Vascular study. We compared expression profiles and conducted pathway analysis using Ingenuity Pathway Analysis (IPA) to reveal differences between ATAA and a healthy artery wall. Almost 5000 genes were differentially expressed in ATAA samples compared to controls. The most downregulated gene was homeobox (HOX) A5 (fold change, FC = −25.3) and upregulated cadherin-2 (FC = 12.6). Several other HOX genes were also found downregulated (FCs between -25.3 and -1.5, FDR < 0.05). 43, mostly inflammatory, canonical pathways in ATAA were found to be significantly (p < 0.05, FDR < 0.05) differentially expressed. The results remained essentially the same when the 6 dissected ATAA samples were excluded from the analysis. We show for the first time on genome level that ATAA is an inflammatory process, revealing a more detailed molecular pathway level pathogenesis. We propose HOX genes as potentially important players in maintaining aortic integrity, altered expression of which might be important in the pathobiology of ATAA.

## Introduction

Ascending thoracic aortic aneurysms (ATAA) are a group of degenerative, often atherosclerotic, inflammation diseases in which the integrity of the aortic wall weakens due to changes in the extracellular matrix (ECM) and a part of the vessel starts to dilate and bulge out forming an aneurysm.

Degenerative aneurysms of the ascending aorta are the most common type of thoracic aortic aneurysms, with incidence of 6–10 per 100 000^1^. The most important risk factor for ATAA alongside with genetic disorders is hypertension^2^. Other important risk factors of ATAA includes smoking, male gender, over 60 years of age^1,3^ and atherosclerosis. ATAA is caused by weakening changes occurring in the aortic wall, especially in the elastic tunica intima and media^4^, in which the amount of elastin decreases and elastin becomes fragmented^5^ thus making the wall more susceptible to bulging out i.e. forming an aneurysm. This type of change predisposing the aortic wall to bulge out is often associated with atherosclerosis^6^. In addition, changes occur in regard to collagen, especially the type IV collagen related to the *vasa vasorum* ^4^. Multiple gene expression changes of important inflammatory and ECM-remodeling machinery genes have been demonstrated between healthy and diseased ascending aorta, for example in the expression of ECM remodeling matrix metalloproteases 2 and 9 (MMP-2 and −9)^7,8^ and a disintegrin and metalloproteinases (ADAMs) 8 and 15^9^. Also, the expression of tissue inhibitors (TIMPs) of these metalloproteases has recently come in to the focus of research in aortic diseases, especially in ATAA research^5^.

The incidence of aortic dissection is less common (0.5–6 per 100 000^1,10,11^) and of these only a minority involves the ascending aorta. Ascending aortic dissection is usually caused by a tearing of ATAA as is the case for the patients in our study. ATAA and aortic dissection can also be caused by rare genetic disorders (e.g., Marfan, Ehlers-Danlos (ED) or Loeys-Dietz (LD), or Turner syndrome), a trauma, an inflammatory disease (autoimmune or microbial based) or the cause may remain unclear^1^.

To our best knowledge this work is the first whole genome wide expression profiling of ATAA. It is important to differentiate the ascending and descending aortic tissues since aorta is not an embryologically uniform structure^12^, as ascending thoracic and abdominal aortas have different developmental origins^12^. In previous works, whole genome profiling has revealed an important role of pathways of the immune system and cell adhesion in the pathogenesis of abdominal aortic aneurysms (AAA)^13,14^. The multifactorial disease of ATAA is regarded as a “silent killer” due to its lack of clinical symptoms before late complications^15^. Several other conditions, including AAA, have been shown as predisposing factors to ATAA^15^. Hence it is crucial to research the processes, which are active and affect these, often lethal and requiring aortic surgery to prevent aortic rupture and dissection.

In this study we aim to describe differences in expression of individual genes and pathways between ATAAs and histologically healthy arterial wall obtained from left internal thoracic artery during cardiac bypass surgery from patients with coronary artery disease.

## Materials and Methods

### Tampere Vascular Study

The samples were collected from patients undergoing cardiac surgery in the Heart Center of Tampere University Hospital with informed consent^9^. Patient information was collected with questionnaires and from the patient database of the hospital. Of the ATAA samples (N = 32, 23 men, 9 women) six (N = 6) had progressed to dissection. The mean age of our cases was 62.6 years and there were no patients with rare genetic disorders (e.g., Marfan, Ehlers-Danlos (ED) or Loeys-Dietz (LD), or Turner syndrome) or a trauma. The control arteries (N = 28) were collected from atherosclerosis-resistant^16^ left internal thoracic artery (LITA) from patients undergoing coronary artery bypass surgery. LITA tissue was chosen, as it is a histologically healthy and is ethically accessible. Demographics of the study population are presented in Table 1. The Ethics Committee of Pirkanmaa Hospital District has approved of this study and the clinical investigation followed the principles of Helsinki declaration.

**Table 1:** Demographics of the study population.

	Ascending aortic aneurysm	Left internal thoracic artery
N	32	28
Age (mean, SD)*	62.6 (12.9)	67.3 (9.6)
Men (%)*	71.9	82.1
BMI (mean kg/m^2^, SD)	28.1 (6.5)	28.9 (5.1)
Smoking (%)*	43.8	64.3
Hypercholesterolemia (%)*	53.1	85.7
Hypertension (%)*	93.8	100.0
Cardiac insufficiency (%)*	34.4	21.4
Statin users (%)*	41.9	82.1
Coronary artery disease (%)*	21.9	100.0
Myocardial infarction (%)*	9.4	39.3
Dissected (aorta) (%)	18.8	—

*Statistically significant difference (p < 0.05). In categorical variables (sex, smoking, hypercholesterolemia, hypertension, cardiac insufficiency, statin user, coronary artery disease and myocardial infarction) p < 0.05 with both chi-square test and Fisher’s exact test. With age and BMI we used independent samples t-test.

### RNA isolation and genome wide expression analysis (GWEA)

The fresh tissue samples obtained from surgery were soaked in RNALater solution (Ambion Inc., Austin, TX, USA) and RNA isolated with Trizol reagent (Invitrogen, Carlsbad, CA, USA) and the RNAEasy Kit (Qiagen, Valencia, CA, USA). The quality as well as concentration of the RNA was evaluated spectrophotometrically (BioPhotometer, Eppendorf, Wesseling-Berzdorf, Germany). Over 23,000 known and candidate genes were analyzed with Illumina HumanHT-12 version 3 Expression BeadChip (Illumina Inc.), following the instructions provided by the manufacturer. (Illumina, San Diego, CA, USA). In brief, 200 ng aliquots of total RNA from each sample were amplified to cDNA using the Ambion’s Illumina RNA Amplification kit according to the instructions (Ambion, Inc., Austin, TX, USA). Samples of cRNA (1500 ng) were hybridized to Illumina’s Expression BeadChip arrays (Illumina). Hybridized biotinylated cRNA was detected with 1 µg/ml Cyanine3-streptavidine (Amersham Biosciences, Piscataway, NJ, USA). BeadChips were scanned with the Illumina BeadArray Reader. The accuracy of this array has been previously tested and in our TVS validation study in which the results of 192 differentially expressed genes were verified by quantitative reverse transcription polymerase chain reaction (qRT-PCR)^17^. The correlation between expression measurements from GWE and qRT-PCR methods was good (r = 0.87, y = 0.151 + 0.586x), and the Bland-Altman plot showed that the fold changes (FCs) were in agreement with these two methods, although for highly up- or down-regulated transcripts, GWE yielding lower absolute FC values than in qRT-PCR.

### Microarray data analysis and pathway analysis

After background subtraction, raw intensity data were exported using the Illumina GenomeStudio software. Raw expression data were imported into R version 3.1.1 (http://www.r-project.org/), log2 transformed and normalized by the locally estimated scatterplot smoothing normalization method implemented in the R/Bioconductor package Lumi (www.bioconductor.org). Locally estimated scatterplot smoothing (LOESS) normalization for the data was selected because it gave the best accuracy in comparison with qRT-PCR data for artery samples^17^. Data quality control criteria included detection of outlier arrays based on the low number of robustly expressed genes and hierarchical clustering.

In order to estimate FC between groups, we calculated differences between medians (in log2 scale) and then back transformed the log ratios to FCs. To make the interpretation easier, we replaced fold-change values that are <1 by the negative of its inverse. Statistical significance of differences in gene expression was assessed using the nonparametric Wilcoxon signed-rank test and the log-transformed data.

Using the “Core Analysis” function of QIAGEN’s Ingenuity Pathway Analysis (IPA, QIAGEN Redwood City, www.qiagen.com/ingenuity) we analyzed the gene expression data to put it in context with the potential biological functions and known pathological mechanisms. The inclusion criteria for genes selected for the analysis was a FC >  ± 1.5, a p-value < 0.05 and an FDR < 0.05 leading to 4884 genes being selected for further analysis. Gene ontology using GO term analysis with GOrilla online tool^18^ was also performed.

## Results

### Differentially expressed genes in ascending thoracic aortic aneurysms in comparison to healthy control arteries

In our ATAA vs. control sample comparison we found a total 4884 significantly differentially expressed genes of which 3115 were up- and 1769 down-regulated (FC >  ± 1.5, p-value < 0.05 and FDR < 0.05). The genes with FC >  ± 5 and FDR < 0.05 are presented in Table 2, and all differentially expressed genes (FC >  ± 1.5 and FDR < 0.05) can be found in Supplementary Table 1. When we excluded the 6 dissected ATAA samples from the analysis, the results remained essentially the same (3228 genes were up- and 1769 genes significantly down regulated).

**Table 2:** The most up- and down-regulated (FC >  ± 5) genes in ascending thoracic aorta aneurysm samples when compared to left internal thoracic artery.

Symbol	Accession	FC	P-value	FDR	Symbol	Accession	FC	P-value	FDR
Down-regulated					Up-regulated				
HOXA5	NM_019102.2	−25.3	4.59*10^−16^	1.37*10^−13^	CDH2	NM_001792.2	12.6	9.18*10^−16^	2.01*10^−13^
ACTA1	NM_001100.3	−17.9	5.51*10^−15^	7.87*10^−13^	CYTL1	NM_018659.2	10.4	9.18*10^−16^	2.01*10^−13^
RGS7BP	NM_001029875.1	−14.7	4.59*10^−16^	1.37*10^−13^	SCG2	NM_003469.3	10.2	9.18*10^−16^	2.01*10^−13^
PTCHD1	NM_173495.2	−13.4	4.59*10^−16^	1.37*10^−13^	IGLL1	NM_020070.2	9.5	2.09*10^−8^	3.42*10^−7^
HOXC6	NM_004503.3	−13.4	4.59*10^−16^	1.37*10^−13^	RPRML	NM_203400.1	8.8	1.71*10^−13^	1.26*10^−11^
LOC285016	NM_001002919.1	−12.1	4.59*10^−16^	1.37*10^−13^	COL21A1	NM_030820.2	8.2	1.84*10^−15^	3.42*10^−13^
HOXC8	NM_022658.3	−11.7	4.59*10^−16^	1.37*10^−13^	LOC652694	XM_942302.1	8.1	5.13*10^−9^	9.77*10^−8^
MUSTN1	NM_205853.2	−11	8.73*10^−15^	1.13*10^−12^	LOC652493	XM_941953.1	7.8	1.59*10^−8^	2.68*10^−7^
RBPMS2	NM_194272.1	−10.4	4.59*10^−16^	1.37*10^−13^	IGJ	NM_144646.2	7.8	9.66*10^−7^	9.86*10^−6^
SEMA3E	NM_012431.1	−8.8	4.59*10^−16^	1.37*10^−13^	C5orf46	NM_206966.2	7.2	1.25*10^−13^	9.77*10^−12^
WFDC1	NM_021197.2	−8.5	6.38*10^−14^	5.65*10^−12^	HAPLN1	NM_001884.2	7.1	4.59*10^−16^	1.37*10^−13^
FZD3	NM_017412.2	−8.3	9.18*10^−16^	2.01*10^−13^	IL6	NM_000600.1	6.9	1.04*10^−5^	7.64*10^−5^
HOXA9	NM_152739.3	−7.8	4.59*10^−16^	1.37*10^−13^	LOC647450	XM_936518.1	6.8	1.39*10^−8^	2.38*10^−7^
HBG2	NM_000184.2	−7.8	3.27*10^−5^	2.05*10^−4^	CTHRC1	NM_138455.2	6.6	9.18*10^−16^	2.01*10^−13^
ALAS2	NM_001037968.1	−7.5	8.09*10^−11^	2.56*10^−9^	SPINT2	NM_021102.2	6.3	3.21*10^−15^	5.14*10^−13^
SRL	NM_001098814.1	−7.5	5.51*10^−15^	7.87*10^−13^	ARPP^−^21	NM_016300.4	6.3	1.25*10^−12^	6.89*10^−11^
LEP	NM_000230.1	−6.8	3.45*10^−10^	9.14*10^−9^	ATP1B1	NM_001677.3	6.2	3.21*10^−15^	5.14*10^−13^
ABCA6	NM_080284.2	−6.7	9.18*10^−16^	2.01*10^−13^	RPLP1	NM_001003.2	5.8	4.59*10^−16^	1.37*10^−13^
UBN2	NM_173569.2	−6.4	4.59*10^−16^	1.37*10^−13^	IL13RA2	NM_000640.2	5.6	3.08*10^−14^	3.06*10^−12^
CIDEC	NM_022094.2	−6.2	2.53*10^−5^	1.64*10^−4^	CAPZA1	NM_006135.1	5.6	4.59*10^−16^	1.37*10^−13^
KIAA1881	XM_001130790.1	−6.1	5.39*10^−6^	4.34*10^−5^	LOC642113	XM_936253.1	5.5	6.99*10^−7^	7.42*10^−6^
FHL5	NM_020482.3	−6.1	9.18*10^−16^	2.01*10^−13^	DNER	NM_139072.3	5.5	3.03*10^−11^	1.09*10^−9^
PCDH17	NM_014459.2	−6	4.59*10^−16^	1.37*10^−13^	RAPH1	NM_213589.1	5.4	5.51*10^−15^	7.87*10^−13^
LOC642678	XM_926130.1	−5.8	4.59*10^−16^	1.37*10^−13^	SPP1	NM_000582.2	5.2	1.05*10^−8^	1.85*10^−7^
BTC	NM_001729.1	−5.8	4.59*10^−16^	1.37*10^−13^					
OR51E2	NM_030774.2	−5.8	4.59*10^−16^	1.37*10^−13^					
ANGPTL5	NM_178127.2	−5.8	1.25*10^−13^	9.77*10^−12^					
P2RX1	NM_002558.2	−5.7	3.21*10^−15^	5.14E*10^−13^					
	XM_378360	−5.5	4.59*10^−16^	1.37*10^−13^					
HOXC4	NM_014620.4	−5.4	4.59*10^−16^	1.37*10^−13^					
PAK2	XM_001126110.1	−5.4	4.59*10^−16^	1.37*10^−13^					
LOC399888	XM_941808.2	−5.2	1.25*10^−13^	9.77*10^−12^					
AAK1	NM_014911.3	−5.2	4.59*10^−16^	1.37*10^−13^					
RORA	NM_002943.2	−5.1	4.59*10^−16^	1.37*10^−13^					
DDX17	NM_006386.3	−5.1	9.18*10^−16^	2.01*10^−13^					
LOC652726	XM_942351.2	−5.1	4.59*10^−16^	1.37*10^−13^					

In genome-wide analysis several homeobox (HOX) genes were amongst the most significantly downregulated in our comparison, especially HOXA5 and HOXC6. A total of 15 different HOX genes were down-regulated (FCs between −25.3 and −1.5) in the samples from ATAA, and one HOX gene (HOXD11) was upregulated with a FC of 1.8 in comparison to controls (Table 2, Supplementary Table 1). HOXA5 was the most down regulated gene in ATAA with a FC of −25.3 in comparison to the healthy control vessel, whilst the most up-regulated gene was cadherin-2 (CDH2; FC = 12.6). Many chemotactic genes were also significantly up-regulated in the ATAA samples as, these included interleukin-6 (IL6), cytokine-like 1 (CYTL1) and secretogranin 2 (SCG2), and several other chemokines that induce chemotaxis of leukocytes to the site of inflammation. Apoptosis- and lipid metabolism-related genes cell death-inducing DFFA-like effector c and a (CIDEC and CIDEA) were also significantly downregulated in our samples.

We also present the results we got when we compared dissected to non-dissected samples in Supplementary Table 2.

### Ingenuity Pathway analysis (IPA) and gene ontology (GO term) analysis

In total 43 canonical pathways were significantly (FDR < 0.05) differentially expressed in ATAA samples in comparison to control arteries (Table 3). Many of the pathways are inflammation-related or involved in the alterations in the ECM and cell to cell adhesions. Hepatic Fibrosis/Hepatic Stellate Cell Activation was the pathway with the most significantly different expression in our samples when compared to the control arteries. GO term analysis yielded no statistically significant results in ATAA analysis with a false discovery rate (FDR) < 0.05 and p-value < 0.05. Fifteen GO terms were differentially expressed with a higher FDR-limit of 0.25 (Supplementary Table 3) and these included many GO terms related to leukocyte migration and tissue remodeling. We also performed analysis in which we excluded the dissected ATAA samples, but as the most significant results remained the same, we present the results of both gene expression and IPA only in the supplementary files (Supplementary Table 4). In otherwise similar pathway analysis, the results remained essentially the same when the 6 dissected ATAA samples we excluded from the analysis.

**Table 3:** The Ingenuity Pathway Analysis results showing the most differentially expressed pathways (FDR < 0.05) in ascending thoracic aorta aneurysm samples when compared to control vessel (LITA).

Ingenuity Canonical Pathways	FDR*	Ratio		Down-regulated	Up-regulated
Hepatic Fibrosis/Hepatic Stellate Cell Activation	7.08*10^−7^	0.339	12/183 (7%)		50/183 (27%)
Adipogenesis pathway	1.02*10^−5^	0.351	16/134 (12%)		31/134 (23%)
Role of Macrophages. Fibroblasts and Endothelial Cells in Rheumatoid Arthritis	4.17*10^−5^	0.272	19/309 (6%)		65/309 (21%)
Axonal Guidance Signaling	4.17*10^−4^	0.241	39/448 (9%)		69/448 (15%)
Integrin Signaling	9.77*10^−4^	0.274	23/219 (11%)		37/219 (17%)
p53 Signaling	0.001	0.324	7/111 (6%)		29/111 (26%)
Signaling by Rho Family GTPases	0.00240	0.259	20/247 (8%)		44/247 (18%)
Leukocyte Extravasation Signaling	0.00537	0.262	19/210 (9%)		36/210 (17%)
Calcium Signaling	0.00617	0.270	23/178 (13%)		25/178 (14%)
ILK Signaling	0.00933	0.260	23/196 (12%)		28/196 (14%)
Clathrin-mediated Endocytosis Signaling	0.00955	0.259	14/197 (7%)		37/197 (19%)
Ephrin Receptor Signaling	0.01	0.264	15/174 (9%)		31/174 (18%)
RhoA Signaling	0.01	0.287	12/122 (10%)		23/122 (19%)
Regulation of Actin-based Motility by Rho	0.0102	0.308	11/91 (12%)		17/91 (19%)
Complement System	0.0102	0.405	1/37 (3%)		14/37 (38%)
Wnt/Ca + pathway	0.0102	0.351	8/57 (14%)		12/57 (21%)
HMGB1 Signaling	0.0102	0.278	6/133 (5%)		31/133 (23%)
Germ Cell-Sertoli Cell Junction Signaling	0.0115	0.260	19/173 (11%)		26/173 (15%)
RhoGDI Signaling	0.0115	0.260	18/173 (10%)		27/173 (16%)
Neurotrophin/TRK Signaling	0.0132	0.316	9/76 (12%)		15/76 (20%)
Xenobiotic Metabolism Signaling	0.0132	0.234	27/286 (9%)		40/286 (14%)
Glucocorticoid Receptor Signaling	0.0138	0.233	19/287 (7%)		48/287 (17%)
Hypoxia Signaling in the Cardiovascular System	0.0170	0.323	6/65 (9%)		15/65 (23%)
HIF1α Signaling	0.0170	0.278	9/115 (8%)		23/115 (20%)
Production of Nitric Oxide and Reactive Oxygen Species in Macrophages	0.0170	0.249	14/193 (7%)		34/193 (18%)
EIF2 Signaling	0.0179	0.247	6/194 (3%)		42/194 (22%)
Assembly of RNA Polymerase I Complex	0.0179	0.667	2/9 (22%)		4/9 (44%)
IL-8 Signaling	0.0240	0.244	13/197 (7%)		35/197 (18%)
mTOR Signaling	0.0295	0.241	9/199 (5%)		39/199 (20%)
VDR/RXR Activation	0.0324	0.295	5/78 (6%)		18/78 (23%)
LXR/RXR Activation	0.0331	0.264	9/121 (7%)		23/121 (19%)
Role of Osteoblasts. Osteoclasts and Chondrocytes in Rheumatoid Arthritis	0.0331	0.233	15/232 (6%)		39/232 (17%)
Actin Cytoskeleton Signaling	0.0355	0.232	19/228 (8%)		34/228 (15%)
Atherosclerosis Signaling	0.0355	0.260	6/127 (5%)		27/127 (21%)
Granulocyte Adhesion and Diapedesis	0.0355	0.243	9/177 (5%)		34/177 (19%)
Role of IL-17F in Allergic Inflammatory Airway Diseases	0.0355	0.341	3/44 (7%)		12/44 (27%)
Pyrimidine Ribonucleotides Interconversion	0.0372	0.375	4/32 (13%)		8/32 (25%)
Huntington’s Disease Signaling	0.0407	0.228	20/241 (8%)		35/241 (15%)
Wnt/β-catenin Signaling	0.0407	0.243	15/169 (9%)		26/169 (15%)
Renal Cell Carcinoma Signaling	0.0417	0.284	7/81 (9%)		16/81 (20%)
Dendritic Cell Maturation	0.0417	0.237	11/190 (6%)		34/190 (18%)
PCP pathway	0.0417	0.302	6/63 (10%)		13/63 (21%)
Antigen Presentation Pathway	0.0417	0.351	2/37 (5%)		11/37 (30%)

*FDR-value calculated by the Benjamini-Hochberg –method.

## Discussion

Here we conducted a large-scale genome wide expression analysis of the molecular determinants of ATAA. Our study addresses the important question of the molecular mechanisms of differentially expressed genes in ATAA, and also using novel bioinformatic Ingenuity Pathway Analysis describing the biological processes relating the differentially expressed genes to each other as functional pathways. Much of the research on aortic aneurysms and gene expression has so far focused on aneurysms of the abdominal aorta^19^. We further confirm many genes previously reported in association with aortic diseases, but also reveal a number of genes previously unreported in association with diseases of the ATAA. Our study offers a novel perspective for the pathobiology ascending thoracic aortic aneurysms, especially regarding the role of HOX genes as most of the HOX genes we found differentially expressed, have not been associated with cardiovascular diseases before. Based on our results it is clear that inflammation remains a crucial part in aneurysm formation and is responsible for many of the structural changes that result in vascular wall weakening and enabling the development of an aneurysm.

Our results are well in accordance with previous results of immune cell response and differential expression of single genes, acquired previously in abdominal aortic aneurysms studies^13,14,19^. These results further affirm that ATAA is an inflammatory condition^20^. However, these similarities must be critically considered as ascending aorta and abdominal aorta are of different embryological origin. Previous studies have also demonstrated changes in calcium-signaling^14^, cell-death^21,22^ and other inflammatory pathways in abdominal aortic aneurysms, similar to those we here report in ATAA. Interestingly, many of these pathways are similar as those we have previously reported in association with atherosclerosis^23^. The results we present here suggest ATAAs to have a strong inflammatory component, which agrees with previous studies conducted with both ATAA^20^ and abdominal aorta aneurysms^14^. As a novel finding we report the differentially expressed HOX genes in aortic pathological changes, differential expression of which has previously been reported only in few instances in association to cardiovascular pathophysiology^24–26^.

HOX genes are a highly conserved^27^ subgroup of homeobox-genes, crucial in embryonic development and spatial body patterning. There is a total of 39 HOX genes^28^, of which 16 were significantly differentially expressed in our data, suggesting these genes have an integral role in the pathogenesis of aortic disease. The only differentially expressed HOXD gene, HOXD11, was upregulated as opposed to 15 different HOXA, -B and –C genes which were downregulated, some of which had several transcripts (namely HOXA9 and HOXC6 had two transcripts). Our results seem to be fairly well in line with previous work of Seo D. *et al*., who have also shown some of the homeobox genes (HOXA5, -C6, -A4, -D4 and –B2) to be dysregulated in atherosclerosis^29^. Nonetheless, most studies related to differential expression of HOX genes are of cancer pathogenesis^30^. There are only a few studies that have shown HOX genes to have a role in cardiovascular diseases^29^ such as abnormal vasculogenesis^24,31^, therefore further research in this area is imperative.

When interpreting these results, we need to keep in mind the site-specificity of HOX gene expression, meaning that HOX gene expression is different in different parts of the healthy aorta, in a collinear manner typical for HOX genes^28,32,33^. The down-regulation of HOXA5 in our samples is in accordance with results from *in vivo* and murine models presented by Arderiu *et al*., which show normal HOXA5 expression to reflect a more stable vascular phenotype^25^. Suppression of HOXA5 has also been shown to be important in angiogenesis^34^ thus giving merit to our results as angiogenesis, induced by leukocytes^35^, is active in inflamed tissues such as atherosclerotic and aneurysmatic vessels. We suggest that the downregulation of HOXC6 is related to the apoptotic activity, as this association has been previously shown by Ramachandran S. *et al*. in human prostate^36^.

Many chemotactic genes were differentially expressed in the ATAA samples, further demonstrating the inflammatory nature of aortic aneurysms^37,38^. Amongst the most upregulated were the genes CDH2, CYTL1 and SCG2. We consider the up-regulation of CDH2 to be merely an indicator of mesenchymal stem cells with the potential to differentiate into cardiac myocytes^39^. The upregulation of secretogranin 2 (SCG2) is a sign of transendothelial migration of leukocytes as shown by Kähler CM. *et al*.^40^ in the ascending aorta, and in this manner we believe it contributes to the loss of integrity in the aortic wall, making the aorta susceptible to aneurysm or even progression to dissection. CYTL1, along with other chemokines such IL6 and SPP1^41^, attract leukocytes which in turn infiltrate the aortic wall and cause other inflammatory effects. This results in enhanced vascular permeability and loss of integrity in the aortic wall^42^, and thereby ensuing in aneurysm formation.

Our results show MMPs 12 and 28 to be differentially expressed with FCs of 2.6 and −2.5 respectively. Many MMPs are associated with cardiovascular diseases, including ATAAs, and particularly ECM-remodeling^43–45^ and as both MMP 12 and 28 are macrophage associated, this further links leukocyte infiltration and function to the changes in the ascending aorta^21^. MMP28 has been implicated in the up keeping of tissue homeostasis by Balta S. *et al*.^46^ and even though it has mostly been investigated in association with various cancers, our results suggest it is related to aortic disease as well, through the same process of dysfunction in tissue homeostasis.

Aortic aneurysms are most commonly located in the abdominal aorta, and due to this, most of the previous similar research has focused on those. The important role of pathways of the immune system and cell adhesion have repeatedly been demonstrated in similar studies of whole genome expression and pathways analysis^13,14,19^. Several similarities can be recognized between these previous works, many inflammatory genes we report here, such as osteopontin (SPP1) and interleukin-6 (IL6), are introduced in these previous works by Hinterseher I. *et al*.^13,19^. It is also noteworthy that the pathways described in these previous works such as Ca^2+^ -signaling^13^ and leukocyte transendothelial migration^14^ are also well in line with our results. Both of the aforementioned and many more similar pathways are also differentially expressed in our samples, but some have slightly different names as the pathway analyses have been conducted with different programs. These similarities in differentially expressed pathways further confirm the role of inflammation to be important in the pathobiology of aortic aneurysms.

The pathways we found differentially expressed suggest that inflammation, leukocyte accumulation and apoptosis are important events occurring in diseased ascending aorta. Pathways we found significantly differentially expressed consist mostly of inflammatory pathways with some hypoxia related signaling as well. We consider the differentially expressed hypoxia induced signaling pathways^47^, together with the differential expression of HOX genes^26^, to show that the processes necessary for the formation of neovessels and repair of the vascular wall are active in the aneurysmatic aorta^48^. Other pathways, that are differentially expressed, are likely to be a result of leukocyte-activity at the aortic site. Pathways such as “Integrin signaling” and “Leukocyte Extravasation Signaling” show that alterations of the arterial wall occur in order to allow leukocytes through, thus further compromising the integrity of the arterial wall^49^ through complex networks of cytokines and other inflammatory mediators, as has been previously studied in both mice^50^ and humans^51^. Our results regarding the p53-signaling in ascending aorta are in line with those of Leeper N. *et al*.^52^ relating this to the apoptosis of smooth muscle cells in the aortic wall^20^. The results we present here support the idea of inflammatory response as the most crucial characteristic of ATAA and show this disease to be an inflammatory condition^20,53^.

### Limitations of the study

The histologically healthy non-atherosclerotic LITA was used as a control vessel. The use of LITA as the control vessel was based on the fact that ethical problems would arise from attempting to collect corresponding healthy aortic samples for gene expression studies during normal surgical operations. Due to LITAs many biologically analogous features we consider it to be the best control readily available^16,54,55^. We acknowledge that especially the inflammatory pathways are to be expected to differ in LITA^56^, as has been shown in methodologically similar study by Ferrari G. *et al*.^56^, but this also does furthermore also support the crucial role of inflammation in ATAA. However, this means we must take in to account the bias caused by LITA control when interpreting these results, especially in regards to the inflammatory pathways being overestimated. We have also used it in our previous studies in comparison of different arterial beds including descending aorta as a control vessel^23^. Our LITA samples have been also collected and analyzed with exactly the same method than ATAA and thus are methodologically ideal controls as in our previous TVS works^23^. To control the effect of ATAA dissection to gene all analyses were performed in two ways with (n = 32) and without dissection (n = 26), with essentially same results. We also emphasize careful interpretation of the results regarding HOX genes, because HOX genes are important in developmental biology and our samples and controls are of different embryological origin.

As many cardiovascular patients have several drugs in use, we must also acknowledge that the use of pharmacologically active agents may affects the gene expression in vascular wall. And as there was a significant difference in statin usage between the cases and controls, this might have an effect on our results. Nonetheless, the differences in medication or in risk factors (i.e. smoking, hypercholesterolemia etc.) are not sufficient to explain the significant differential expression of genes we have presented here and thus we consider our results to be of significance in furthering our understanding the pathobiology of ATAA.

## Conclusions

Our findings expand the current understanding of ATAA pathogenesis on the molecular level. Our results show for the first time at the genome level that ATAA is an inflammatory process, and pinpoint its detailed molecular and pathway level pathogenesis. We also propose cautiously HOX genes as possibly crucial players in maintaining aortic integrity, and altered expression of these transcription factors may lead to susceptibility to pathological changes in the ATAA and even lead to a need for aortic surgery. This however might, at least partially, be due to the different embryological origin of the compared vessels. Nonetheless our result well depict important molecular pathways that are significant in ATAA pathogenesis and offer a wide range of possibilities for future research on this serious, yet relatively little studied, disease.

## Electronic supplementary material

Supplementary information

Supplementary table 1

Supplementary table 2

Supplementary table 3

Supplementary table 4
