# Supplementary material for: Differentially expressed genes and canonical pathways in the ascending thoracic aortic aneurysm – The Tampere Vascular Study
Source: Sci Rep. 2017 Sep 21;7:12127. doi: 10.1038/s41598-017-12421-4 (PMC5608723; doi:10.1038/s41598-017-12421-4)
Supplement: Supplementary file 1 — Supplementary information [file 41598_2017_12421_MOESM1_ESM.pdf]

Differentially expressed genes and canonical pathways in the ascending thoracic aortic aneurysm – The Tampere Vascular Study

Miska Sulkava[1*], BM; Emma Raitoharju[1], PhD; Ari Mennander[2], MD, PhD; Mari Levula[1], PhD; Ilkka Seppälä[1], MSc, BM; Leo-Pekka Lyytikäinen[1], MD; Otso Järvinen[2], MD, PhD; Thomas Illig[3, 4, 5], MD, PhD; Norman Klopp[3, 4], PhD; Nina Mononen[1], PhD; Reijo Laaksonen[1], MD, PhD; Mika Kähönen[6], MD, PhD; Niku Oksala[1,3], MD, PhD, DSc; Terho Lehtimäki[1], MD, PhD.

Supplementary information

Supplementary Table 1: The genes differentially expressed when comparing ATAA samples to control vessels.

Supplementary Table 2: The genes differentially expressed in dissected ATAA samples when compared to non-dissected ATAA samples.

Supplementary Table 3: The results from GO-term analysis.

Supplementary Table 4: The IPA results when the dissected samples were excluded.
